# Genome Partitioner: A web tool for multi-level partitioning of large-scale DNA constructs for synthetic biology applications

**DOI:** 10.1371/journal.pone.0177234

**Published:** 2017-05-22

**Authors:** Matthias Christen, Luca Del Medico, Heinz Christen, Beat Christen

**Affiliations:** 1 Institute of Molecular Systems Biology, Department of Biology, Eidgenössische Technische Hochschule (ETH) Zürich, CH-8093 Zürich, Switzerland; 2 Life Science Zürich PhD Program on Systems Biology, Zürich, Switzerland; University of Helsinki, FINLAND

## Abstract

Recent advances in lower-cost DNA synthesis techniques have enabled new innovations in the field of synthetic biology. Still, efficient design and higher-order assembly of genome-scale DNA constructs remains a labor-intensive process. Given the complexity, computer assisted design tools that fragment large DNA sequences into fabricable DNA blocks are needed to pave the way towards streamlined assembly of biological systems. Here, we present the Genome Partitioner software implemented as a web-based interface that permits multi-level partitioning of genome-scale DNA designs. Without the need for specialized computing skills, biologists can submit their DNA designs to a fully automated pipeline that generates the optimal retrosynthetic route for higher-order DNA assembly. To test the algorithm, we partitioned a 783 kb *Caulobacter crescentus* genome design. We validated the partitioning strategy by assembling a 20 kb test segment encompassing a difficult to synthesize DNA sequence. Successful assembly from 1 kb subblocks into the 20 kb segment highlights the effectiveness of the Genome Partitioner for reducing synthesis costs and timelines for higher-order DNA assembly. The Genome Partitioner is broadly applicable to translate DNA designs into ready to order sequences that can be assembled with standardized protocols, thus offering new opportunities to harness the diversity of microbial genomes for synthetic biology applications. The Genome Partitioner web tool can be accessed at https://christenlab.ethz.ch/GenomePartitioner.

## Introduction

The ability to assemble DNA into single and multipart constructs has propelled molecular biology since the discovery and first use of restriction endonucleases over 40 years ago [1]. The past decade has seen several technological improvements that shaped the field of synthetic biology and enabled the design and construction of new biological parts and modules up to the size of genome-scale biological operating systems [2]. Foremost, this was made possible by rapid advances in lower-cost, high fidelity DNA synthesis techniques [3]. Commercial gene synthesis is now offered from numerous companies that provide sequence confirmed constructs up to 4 kb in size. In addition, biological design standards such as BioBricks [4], Golden Gate Cloning [5], BASIC [6] and many others have been created to assist modular assembly of DNA parts into higher order constructs [7,8]. More recently, isothermal assembly and *in vivo* recombineering techniques have removed many of the bottlenecks surrounding the assembly of large DNA fragments by classical restriction/ligation methods. Such overlap-based assembly methods can easily assemble five or more DNA parts together in a one step process [9]. Thereby, an array of smaller DNA fragments is linked together in an end-to-end fashion, either by joining of short overlapping sequences by *in vitro* isothermal assembly methods [10], or by transformation of linear DNA parts into *Saccharomyces cerevisiae* and use of its homologous recombination DNA repair machinery [11,12].

Even with the simplicity of homologous end joining methods, navigating the design and build process for multi kilo-base to mega-base synthetic DNA constructs is still a limiting factor for most laboratories. Limitations exist in the fact that not every DNA sequence is accessible through low-cost *de novo* DNA synthesis and commercial providers may reject sequences that are difficult to manufacture. Disfavored sequence features that decrease efficiency of DNA synthesis and assembly include secondary structures and sequence stretches of elevated extreme GC content. Thus, larger DNA designs require computational sequence refactoring prior synthesis by using computational design algorithms [13,14].

The process of partitioning is more complex than just splitting DNA into series of shorter fragments. Additional sequences that guide the assembly process have to be inserted in a nested manner on both ends of the partitioned fragments. These include homology regions between adjacent fragments, specific adaptor sequences needed for cloning into propagation vectors and type IIS restriction sites for subsequent release of fragments. Furthermore, homology regions should not contain DNA secondary structures as these features compete with single-stranded annealing of neighboring assembly fragments, reducing yields during assembly reactions. The larger the DNA designs, the more important is the optimization of homology regions between adjacent DNA blocks. The partitioning process can be seen as a retrosynthetic technique for optimizing the synthesis and assembly of complex genome-scale DNA designs. Given its complexity, manual sequence partitioning of DNA designs is not feasible. Thus, computer-assisted design tools that fragments large DNA sequences into smaller, fabricable DNA blocks are needed to pave the way towards streamlined assembly of biological systems. To address these needs, we have developed the Genome Partitioner software implemented as a web-tool interface that permits multi-level partitioning of large-scale DNA constructs for synthetic biology applications.

## Materials and methods

### The Genome Partitioner web tool

The algorithm is implemented in Python programming language (https://python.org) and utilizes the Biopython package [15] built-in functions for parsing GenBank files and the GenomeDiagram plotting routines [16] of Biopython’s Bio Graphics package to generate graphic output files of the partitioning design. The Genome Partitioner web tool provides a detailed online documentation including explanation of partitioning parameters, supported partitioning modes as well as a descriptions of log files, sequence output files and statistics files.

**Fig 1:**
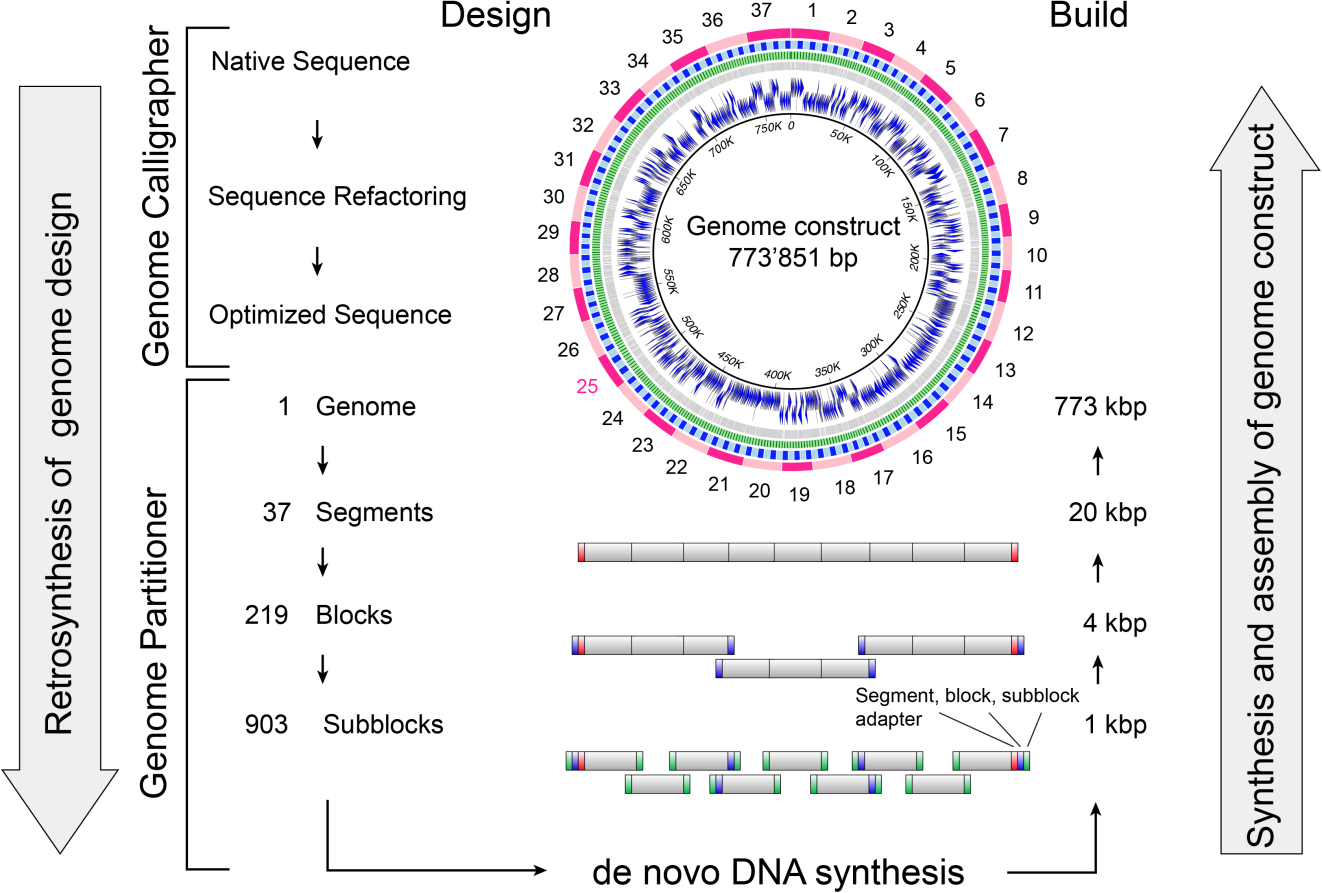
Flowchart illustrating the design-to-build workflow for the streamlined assembly of genome-scale DNA constructs. In a first retrosynthesis step, the native sequence GenBank file is optimized for *de novo* DNA synthesis using the Genome Calligrapher algorithm. Next, retrosynthetic partitioning by the Genome Partitioner translates the genome design into ready to order 1 kb subblocks. Thereby homology regions are computationally optimized and nested adapter sequences that guide subsequent assembly steps are inserted on both ends of the partitioned fragments. *De novo* DNA synthesis of genome constructs begins with manufacturing of 1 kb subblocks by a commercial provider of *de novo* DNA synthesis. Subsequent higher order assembly follows the standardized, four-tier synthesis strategy generated by the Genome Partitioner algorithm.

The algorithm generates up to four assembly tiers (Fig 1). At the lowest assembly tier, the Genome Partitioner generates 1 kb subblock sequences in FASTA format to be chemically synthesized by *de novo* DNA synthesis. Using overlap-based DNA assembly methods, these subblocks can be joined into intermediate 4 kb DNA assembly units (termed blocks) that are further assembled into 20 kb DNA assembly units (termed segments). At the highest assembly tier, DNA segments are assembled into the desired genome-scale DNA construct. For each assembly tier, the algorithm generates nested adaptor sequences consisting of homology regions for seamless recombination-based *in vitro* and *in vivo* assembly and cloning of adjacent units. Prefix (5') and suffix (3') adapter sequences that contain homologies for the integration of assemblies into maintenance vectors and type IIS restriction sites for the subsequent release are added to each assembly unit (Fig 1 and S1 Fig). Adapter sequences from higher assembly tiers are programmed into assembly units from lower tiers. A separate GenBank output file annotates subblocks, blocks and segments of the partitioned DNA design. It provides the blueprint for quality control during *de novo* DNA synthesis and subsequent higher order DNA assembly.

### The partitioning workflow

#### GenBank file upload

To start the partitioning design process, the user first specifies a single GenBank file for server upload at the web interface front page. The input sequence must be smaller than 10 Mb in file size. Splitting of the DNA design into smaller sequences is recommended when processing larger input files. The Genome Partitioner algorithm is primarily intended for the partitioning of prokaryotic genome-scale DNA constructs. However, DNA designs derived from eukaryotic sequences can also be processed as long as no discontinuous features or long interspersed repeat sequences are present.

#### Partitioning parameter settings

**Fig 2:**
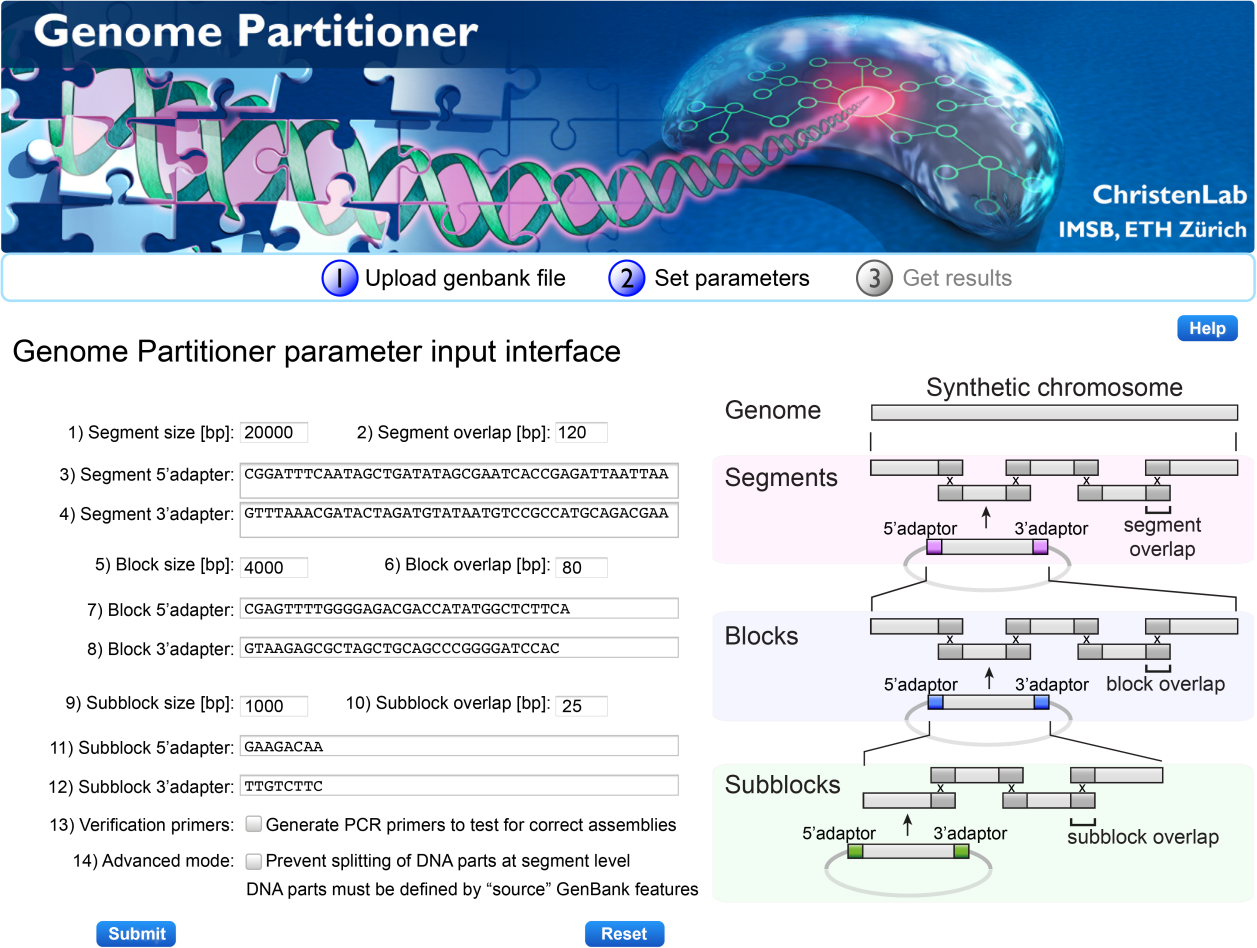
Overview of the Genome Partitioner web interface. In the left panel, the parameter input interface allows the user to customize partitioning optimization criteria, to specify size and homology overlaps of assembly units, and define preset or customized adaptor sequences. The user can optionally generate specific PCR primers to test for correct assemblies at the block, segment and genome level and select between an equidistant partitioning and an advanced mode where the partitioning at the segment level follows the boundaries of biological parts. The right panel provides a flowchart illustrating the four-tier assembly workflow. Assembly units of increasing size are formed using overlap-based DNA assembly methods.

After upload, a new graphic interface appears (Fig 2) where the user defines partitioning specific parameters. Direct and inverted repeat sequences and non-unique sequence stretches within terminal homology regions can lead to misalignment and imprecise end joining of assembly units resulting in short sequence deletions, duplications or formation of non-desired assembly side products. The Genome Partitioner algorithm uses build in rules to generate terminal homology regions between adjacent assembly units that are devoid of sequences that impede recombination-based homologous end joining. Build-in parameter settings are used for the detection of sequence features such as direct repeats, hairpin structures and non-unique sequence stretches that occur in multiple terminal homology regions (Table 1). For each assembly tier, the user can specify the maximal size in base pairs for segments (input field 1), blocks (input field 5) and subblocks (input field 9). This is in particular useful for optimization of synthesis costs, when restrictions in sequence length apply. The maximal unit size is defined as the partition sequence plus flanking 5’ prefix and 3’ suffix adapter sequences derived from current and subsequent higher order assembly tiers. Further, the size of terminal homology regions (termed 'overlaps') can be specified for segments (input field 2), blocks (input field 6), and subblocks (input field 10). Overlaps of 25 bp are sufficient at the lowest tier where *in vitro* assembly methods are preferentially used for homologous end joining of subblocks. At higher tiers of block and segment assembly where yeast homologous recombination is the method of choice, preset values for overlaps are 80 bp and 120 bp respectively.

**Table 1:** List of the built-in optimization parameters used by the Genome Partitioner algorithm.

Sequence feature	Impaired DNA assembly due to:	Size limit
Direct repeats	Mis-alignment of overlap sequences leading to short deletions and insertions	8 bp (built-in)
Hairpins	Formation of secondary structures that impede proper annealing of overlap sequences	8 bp (built-in)
Non-specific overlaps	Mis-annealing of overlap sequences leading to undesired assembly product	8 bp (built-in)

#### Design of adapter sequences

Further, the user can select preset or specify customized 5’ prefix and 3’ suffix adapter sequences that permit the integration of assembly units into corresponding destination vectors (input fields 3,4,7 and 8). Adapter sequences also contain recognition sequences for type IIS restriction enzymes that facilitate the release of assembly units from cloning vectors. The predefined adaptor sequences contain all necessary type IIS recognition sites and can be used for the assembly of blocks into pXMCS-2 and segments into pMR10Y cloning vectors. At the level of subblocks, a single type IIS recognition site such as the predefined adaptor for BbsI is sufficient (input fields 11 and 12) for the release of subblocks from plasmid cloned DNA. If subblocks are provided in form of linear DNA, no 5' prefix and 3' suffix adaptor sequences are required and input fields 11 and 12 remain empty. When designing adapter sequences, the user must select restriction sites that are absent in the DNA construct. The predefined adaptor sequences are BbsI for subblocks, BspQI for blocks, PacI and PmeI for segments. To assist in the removal of internal restriction sites, genome-scale DNA design tools such as the Genome Calligrapher web tool [13] can be used (https://christenlab.ethz.ch/GenomeCalligrapher).

#### Primer design for PCR verification of assemblies

In input field 13, the users can optionally generate sets of diagnostic PCR primers that allow for the testing of correctly assembled constructs and sequence verification across assembly junctions. The Genome Partitioner algorithm calculates suitable PCR primer-pairs surrounding the assembly junctions of segments, blocks and subblocks. For each assembly junction, forward and reverse primers, each 20 bp in size, are generated that anneal within a pre-defined search window of 150 bp flanking the terminal homology region. PCR primers are analyzed to define suitable primer pairs that pass the following design-rules: 1) Each primer pair must fall within a predefined melting temperature interval (∆Tm < 1°C). 2) The sequence of the last 8 bases from the 3’ end of each primer must be unique within the search window. 3) Primer pairs show no homologies and self-homologies. 4) Each primer must be devoid of hairpins and repeats larger than 4 bases. For each assembly junction, the first pair of primers passing the design rules is written into the corresponding output files segment_primers.txt, block_primers.txt and subblock_primers.txt. If no suitable primer-pair is found, the program will increase the search window by 50 bases followed by increasing the annealing temperature range by 1°C using a maximum of six iterations. If still no primer-pair is found, then the unresolved region is reported in the output file for separate processing. A statistics output of the primer design steps is written in the log file (logfile.txt), showing the number of discarded primer pairs and a description of the optimization problems encountered.

#### Annotation of biological parts

In addition to the default mode, which produces equidistant partitioning designs at the segment level, partitioning can be performed on advanced mode where segment boundaries follow boundaries of biological parts (input field 13). The advanced mode prevents splitting of biological parts into different segments, which is particularly useful for grouping together biological functions on the level of segments. Partitioning in the advanced mode requires biological parts to be annotated using the feature type 'source' of the GenBank format.

FEATURES             Location/Qualifiers

            source           1..182

                                /mol_type = "genomic DNA"

                                /note = "synthetic part:1"

                                /organism = "Caulobacter syntheticus v3_19”

            source         183..2484

                                /mol_type = "genomic DNA"

                                /note = "synthetic part:2"

                                /organism = "Caulobacter syntheticus v3_19"

            source         2485..4847

                                /mol_type = "genomic DNA"

                                /note = "synthetic part:3"

                                /organism = "Caulobacter syntheticus v3_19"

#### Partitioning algorithm

The partitioning algorithm uses a four steps process. In the first step, segments not exceeding a user-defined size limit are generated. The algorithm restrains the variation of the segment sizes to less than 10%. Each segment shares terminal homology regions (called THR hereafter) to adjacent segments. The length of THRs at the segment level is user-defined (in the range of 35 to 200 bp). Segments are flanked by user-defined, segment specific 5’ and 3’ adapter sequences.

In the second step, all segments are further subdivided into blocks with a user-defined size limit (in the range of 2000 to 5000 bp). The size limit of blocks is set not to exceed a size variation larger than 10%. To maximize the size of each block, each segment is divided into the least number of blocks. If necessary, the size of blocks is adjusted to accommodate for subsequent addition of 5’ and 3’ adapter sequences. Blocks that overlap with termini of segments also carry the appropriate adapter sequences of segments. Each block shares THRs with adjacent blocks. The length of THRs at the block level is user-defined (size range between 15 to 200 bp).

For all block THRs of a given segment, the Genome Partitioner algorithm analyses the occurrence of unfavorable secondary structures and sequence patterns prone to interfere with the recombination based DNA assembly process. In a first optimization routine, hairpins and direct repeats are identified within THRs. The maximal length of direct and indirect repeats within THRs must be smaller than 8 bp. If larger repeats are detected, the algorithm generates alternative block partitioning variants with terminal homology sequences shifted up or downstream of the original partitioning site. In a second optimization routine, the algorithm searches for identical substrings occurring multiple times (i.e. non-unique sequences) within THRs of DNA blocks from each segment. Non-unique sequences within THRs larger than 8 bp are removed by generating a set of partitioning variants that no longer include non-unique sequence substrings. These partitioning design variants are iteratively evaluated for occurrence of repeat, hairpins and non-unique sequence substrings within multiple THRs. A metric is used to identify the optimal partitioning design variant that i) shows absence of repeats and ii) is unique in sequence and iii) requires the least repositioning of THR regions. The THR optimization procedure follows a non-recurring search path until repeat sequences and non-unique sequence substring larger than 8 bp have been removed. If repeat sequences, hairpins and non-unique sequence substrings remain after 20 iteration cycles, the last position of THRs is written in the output file.

In the third step, each block of the partitioning design is further subdivided into subblocks. Subblocks deviate by less than 10% from a user-defined size limit (in the range 500 to 2000 bp) including the length of any adapter sequences. To maximize the size of each subblock, each block is divided into the least number of subblocks. If necessary, the size of subblocks is adjusted to accommodate for subsequent addition of 5’ and 3’ adapter sequences. The length of terminal homology regions at the subblock level is user-defined (in the range of 15 to 200 bp). THRs of DNA subblocks are optimized according to the same procedure as used for the THR optimization of blocks. Similar to blocks, subblocks harbor adapter sequences for higher-order assembly. Subblocks that correspond to termini of segments carry composite adapter sequences composed of the innermost segment adapters, followed by the block adapters and the outermost subblock adapter sequences. Similarly, subblocks that upon assembly form the termini of blocks carry composite adapters composed of inner block adapter and outer subblock adapter sequences. All other subblock types only carry subblock adapter sequences. The addition of composite adapter sequences permits release and subsequent assembly of subblocks into higher order assembly units.

In the fourth step, sequences of segments, blocks and subblocks including 5’ and 3’ adapter sequences are written into FASTA files and annotated into the GenBank output file. While smaller DNA constructs, less than 100 kb in size, are partitioned within seconds, larger genome designs may take a few minutes to process. During the partitioning process and overlap optimization, a progress bar shows the percentage of completed assembly units for each partitioning level.

#### Download of partitioning results in FASTA and GenBank file format

After completing the partitioning optimization, the user is relocated to a graphical output interface where FASTA files for assembly units and a GenBank file with the annotated partitioning design can be downloaded (S2 Fig). Downloaded FASTA sequence files can be directly submitted to a commercial provider of *de novo* DNA synthesis. In addition, partitioning parameter settings, statistics and primer files for the verification of DNA assemblies are provided for download. A graphic map of the generated four-tier assembly design is shown. A table summarizes the size range of assembly units and the effective DNA synthesis effort needed for each assembly tier. Furthermore, a comparison of assembly constraints prior and after the optimization is shown (S2 Fig).

### In silico analysis of assembly feasibility

To assess the frequency of sequence patterns that impede large-scale DNA assembly of bacterial genomes, we processed a total of 4967 microbial chromosomes and plasmids with the Genome Partitioner algorithm. Bacterial GenBank sequence files were downloaded from NCBI [17]. Standard partitioning parameter settings were used for the analysis with the default-partitioning mode enabled. Segment, block and subblock sizes were set to 20’000, 4’000 and 1’000 bp respectively. The size of segment, block and subblock overlaps was set to 120, 80, and 35 bp, and the size limits for repeat and non-unique sequence string within overlaps was set to 8 bp. Partitioning was carried out once without optimizing terminal homology regions and once with optimization by the Genome Partitioner algorithm enabled. For each analyzed chromosome, a set of partitioning metrics was calculated including, the number of generated segments, blocks and subblocks, mean, minimal and maximal size of assembly units, the length of each sequence and the length of partitioned sequences including appended adapter sequences. Furthermore, the occurrence of assembly constraints including repeats, inverted repeat and non-unique sequences occurring within assembly overlaps was analyzed. The number of segments with and without any assembly constraints at the block and subblock level was recorded. For each genome processed, the global GC content and the GC content of the subset of segments with and without assembly constraints were calculated using Bio-Python’s build in functions. The detailed data output from the genome-wide partitioning analysis is listed in Data SI.

### In silico design of a synthetic genome

To test the utility of the assembly design process of the Genome Partitioner, we probed the assembly efficiency of a synthetic genome construct composed of essential and high-fitness genes of the cell-cycle model organism *Caulobacter crescentus*. The comprehensive list of DNA parts corresponding to the entire set of essential and high-fitness sequences required for rich-media growth of *Caulobacter crescentus* was generated using a previously identified essential genome data set [13]. The part list includes DNA sequences encoding proteins, RNA and regulatory features as well as small essential inter-genic sequences. Part boundaries of protein coding genes were set to the CDS coordinates according to the *Caulobacter crescentus* NA1000 genome annotation (NCBI Accession: NC_011916.1) plus additional regulatory and terminator regions. Coordinates of regulatory upstream sequences of essential genes were set according to previously identified essential promoter regions and, when necessary, were enlarged to include strong transcriptional start sites as determined by RNAseq [18]. For essential or high-fitness genes, predicted Rho-independent terminator sequences were included as identified by the WebGeSTer DB [19]. The resulting DNA parts were concatenated in order and orientation of the wild-type genome into a 773851 bp long synthetic genome construct. To optimize the sequence for *de novo* DNA synthesis, protein-coding sequences were refactored by neutral recoding (synonymous codon replacement) using the Genome Calligrapher web tool (https://christenlab.ethz.ch/GenomeCalligrapher) [13]. The average recoding probability across segments was set to 0.57, resulting in introduction of 133’354 base substitutions across the 773’851 bp genome design. The first four amino acids codons of each CDS were excluded from recoding to maintain potential translational and other regulatory signals. Disallowed sequences that were removed upon recoding included endonuclease sites for BsaI, AarI, BbsI, BspQI, PacI and PmeI, SceI and CeuI. Furthermore, the AGT, ATA, AGA, GTA and AGG codons, which are rare codons in *Caulobacter crescentus*, were set as immutable codons (neither replaced or introduced upon recoding). The amber stop codons TAG and the two TTA and TTG codon for leucine were erased upon recoding. Occurrence of homopolymeric sequences and di and tri-nucleotide repeats were removed (less than six G, eight C’, nine A or T, less than 10 dinucleotides repeats, less than 6 trinucleotides repeats). Similarly, direct and indirect sequence repeats larger than 11 bp were removed from within CDS using synonymous recoding. The resulting genome design was then partitioned by the Genome Partitioner algorithm into thirty-eight 20 kb long segments that were further partitioned into 4 kb DNA building blocks. Out of them, we selected one chromosome segment (segment 25) to test the assembly feasibility of 1 kb subblocks into 4 kb blocks and subsequently into the 20 kb chromosome segment.

### DNA assembly

#### PCR amplification of subblocks

Subblocks were ordered from a commercial provider of *de novo* DNA synthesis (Gen9, Inc. Cambridge, MA, USA) and provided as sequence confirmed plasmid cloned constructs. Subblock sequences were PCR amplified from maintenance vectors (pG9m-2) in a 25 μl reaction volume using 0.25 μl (2.5 u) Phusion® high-fidelity DNA polymerase (New England Biolabs, USA), 5 μl 5x Phusion® HF reaction buffer (NEB), 0.25 μl (~ 25 ng) plasmid template DNA, 0.125 μl 100 μM forward primer (#1) (S1 Table), 0.125 μl 100 μM reverse primer (#2), 2.5 μl dNTPs (2 mM each) (Thermo Fisher Scientific Inc., USA), 0.75 μl DMSO (Fisher Scientific, UK), and 16 μl ddH_2_O. The PCR reaction was run on a BIORAD S1000 thermal cycler (Bio-Rad Laboratories Inc., USA) using the following protocol. (1) Initial denaturation 3:00 min at 95°C, (2) denaturation 30 s at 95°C, (3) primer annealing 30 s at 58°C, (4) elongation 1:30 min at 72°C, (5) repeat steps 2–4, 25 times, (6) final elongation 5 min at 72°C.

#### Digestion of subblocks and pXMCS-2 target vector

To release subblocks from adapter sequences, PCR products were digested with BbsI type IIS restriction enzyme. Digestion reactions contained four times 2.5 μl of each of the four subblocks directly taken from the PCR reaction mixture, 0.5 μl (5 u) BsbI type IIS restriction enzyme (NEB, USA), 2 μl 10x NEBuffer 2.1 (NEB, USA), and 7.5 μl nuclease-free H_2_O (Promega, USA). The digestion reactions were incubated at 37°C overnight and subsequently column purified using the NucleoSpin® Gel and PCR clean up Kit (Macherey-Nagel, Switzerland). The pXMCS-2 maintenance vector [20] was digested with NdeI and NheI-HF restriction enzymes in a 40 μl digestion reaction volume composed of 20 μl (294.4 ng/μl) pXMCS-2, 0.5 μl (10 u) NdeI (NEB, USA), 0.5 μl (10 u) NheI-HF (NEB, USA), 4 μl 10x CutSmart® buffer (NEB, USA), and 15 μl nuclease-free H_2_O (Promega, USA). The digestion reaction was incubated at 37°C for 4 h. The digested vector was separated on a 1% agarose gel (UltraPure™ Agarose, Invitrogen, USA) and run for 40 min at 120 V, extracted from the gel and eluted in 20 μl using the NucleoSpin® Gel and PCR clean up Kit (Macherey-Nagel, Switzerland).

#### DNA assembly of subblocks into blocks

The BbsI-digested subblocks were assembled into corresponding blocks and integrated in maintenance vector pXMCS-2 (S2 Table) using isothermal assembly reactions consisting of: 4 μl 5x isothermal reaction buffer, 0.008 μl (0.08 u) T5 exonuclease (NEB, USA), 0.25 (2.5 u) Phusion® high-fidelity DNA polymerase (NEB, USA), 2 μl (80 u) Taq DNA Ligase (NEB, USA), 8.742 μl nuclease-free H_2_O (Promega, USA), 4 μl (~ 20 ng/μl) of purified BbsI-digested subblock pool, and 1 μl (108 ng) of NdeI and NheI-HF digested pXMCS-2 vector. Ligation reaction mixtures were incubated at 50°C for 1 h. 5 μl of each of the pXMCS-2::block[0–4] assemblies were dialysed on 0.025 μm VSWP MF™ membrane filters (Merck Millipore Ltd., IRL) for 20 min and subsequently electroporated into competent *E*. *coli* DH5α (90 μl aliquots, OD ~ 15) at 1.75 kV, 400 Ω, and 25 μF using 0.1 cm electrode gap Gene Pulser® cuvettes (Bio-Rad Laboratories, USA). The pulse was applied at time constants between 8.6 and 8.8 ms. Immediately after the electroporation, transformed DH5α cells were rescued in 1 ml SOC medium and incubated at 37°C for 1 h. 100 μl of each rescued cell sample cells was plated onto selective LB medium supplemented with kanamycin (20 μg/ml) and incubated at 37°C overnight.

#### Verification of block assemblies by PCR

Correct block assemblies were verified by PCR of subblock junctions using primer sets generated by Genome Partitioner algorithm. PCR reactions were performed in 20 μl reaction volumes composed of 10 μl 2x GoTaq® G2 Green Master Mix (Promega, USA), 0.5 μl 100 μM forward primer (fw primers of #3–32) (S1 Table), 0.5 μl 100 μM reverse primer (rv primers of #3–32), 1 μl DH5α pXMCS-2::block[0–4] liquid culture, and 8 μl ddH_2_O. The PCR protocol consisted of (1) initial denaturation 3:00 min at 95°C, (2) denaturation 30 s at 95°C, (3) primer annealing 30 s at 60°C, (4) elongation 30 s min at 72°C, (5) repeat steps 2–4, 25 times, (6) final elongation 5 min at 72°C.

#### BspQI-mediated block release

The pXMCS-2::block[0–4] plasmids were isolated from DH5α using the GeneJET Plasmid Miniprep Kit (Thermo Scientific, USA). Subsequently, blocks were released from the pXMCS-2 backbone via BspQI restriction digestion. Each digestion reaction contained 10 μl (> 5 μg) pXMCS-2::block[0–4] plasmid, 1μl (10 u) BspQI type IIS restriction enzyme (NEB, USA), 4 μl 10x NEBuffer 3.1 (NEB, USA), and 25 μl nuclease-free H_2_O (Promega, USA). The digestions were incubated at 50°C for 1.5 h and the reactions was stopped by heat inactivation at 80°C for 20 min. Subsequently, digested constructs were purified over column using the NucleoSpin® Gel and PCR clean up Kit (Macherey-Nagel, Switzerland).

#### DNA assembly of blocks into segment 25

Purified pXMCS-2::block[0–4] digestion mixtures were used for assembly of segments into yeast vector pMR10Y (pMR10::ARS::URA3). *S*. *cerevisiae* strain VL6-48N (BC3347) was grown in YPD medium supplemented with 80mg/L adenine to OD_600_ of 0.7. 2 ml of the yeast culture was pelleted, washed with 1 ml 0.9% NaCl-solution and pelleted again. 10 μl of fish sperm DNA (10μg/μl, single stranded from salmon testes, D7656, Sigma-Aldrich, USA) was added to the cells. Subsequently, 540 ng of linearized pMR10Y (digested with PacI and PmeI) and 300 ng of each of the five BspQI digested DNA block (block[0–4]) were added to the pellet. After thorough vortexing, the pellet was resuspended in 500 μl transformation mixture composed of 400 μl 50% PEG solution, 50 μl 1M Lithium acetate, 50 μl ddH_2_O. Next, 57 μl DMSO were added to the transformation reaction followed by 15 min incubation at RT and 15 min at 42°. Transformed cells were pelleted, resuspended in 100 μl ddH_2_O and plated onto yeast drop-out medium lacking uracil, supplemented with glucose (10 g/L), adenine (80 mg/L) and incubated at 30°C for three days till colonies appeared.

#### Verification of segment assemblies by PCR

Correct segment assembly was verified by PCR amplifying each block junction using the Genome Partitioner's automatically designed primers. Six colonies were picked and resuspended in liquid yeast synthetic drop out medium lacking uracil and supplemented with glucose (10 g/L) and adenine (80 mg/L). Colony PCR was performed in a 20 μl reaction volume containing 10 μl 2x Phire Green Hot Start II PCR Master Mix (Thermo Scientific, USA), 0.5 μl 25 μM forward primer (fw primers #33–40), 0.5 μl 25 μM reverse primer (rv primers #33–40), 1μl yeast liquid culture, and 8 μl ddH_2_O. The PCR protocol consisted of (1) initial denaturation 3:00 min at 98°C, (2) denaturation 5 s at 98°C, (3) primer annealing 5 s at 62°C, (4) elongation 20 s min at 72°C, (5) repeat steps 2–4, 40 times, (6) final elongation 1 min at 72°C.

## Results and discussion

### DNA-assembly constraints across microbial genomes

To globally examine the degree of partitioning constraints across all sequenced bacterial genomes, we carried out bioinformatics analyses of all completed bacterial genomes deposited at NCBI GenBank database. For each of the 4967 microbial genomes and plasmid sequences, we calculated the fraction of the genome sequence amenable to a four-tier assembly strategy composed of 900 bp subblocks, joined into 3.4 kb blocks and further assembled into 20 kb chromosome segments. Using equidistant partitioning, we calculated the occurrence of repetitive sequences larger than 8 bp within terminal homology regions. Furthermore, to exclude undesired assembly side products, we required homology regions to have less than 8 bp in common with other units (S1 File). Our *in silico* analysis revealed that not a single of the 4967 genome sequence tested could be assembled in a risk-free manner using equidistant partitioning. Cumulatively, on the level of 20 kb segments, only 30.9% of all NCBI’s microbial genome sequences are readily amenable to higher-order assembly, while a large fraction of 69.1% cannot be reliably built (S1 File).

**Fig 3:**
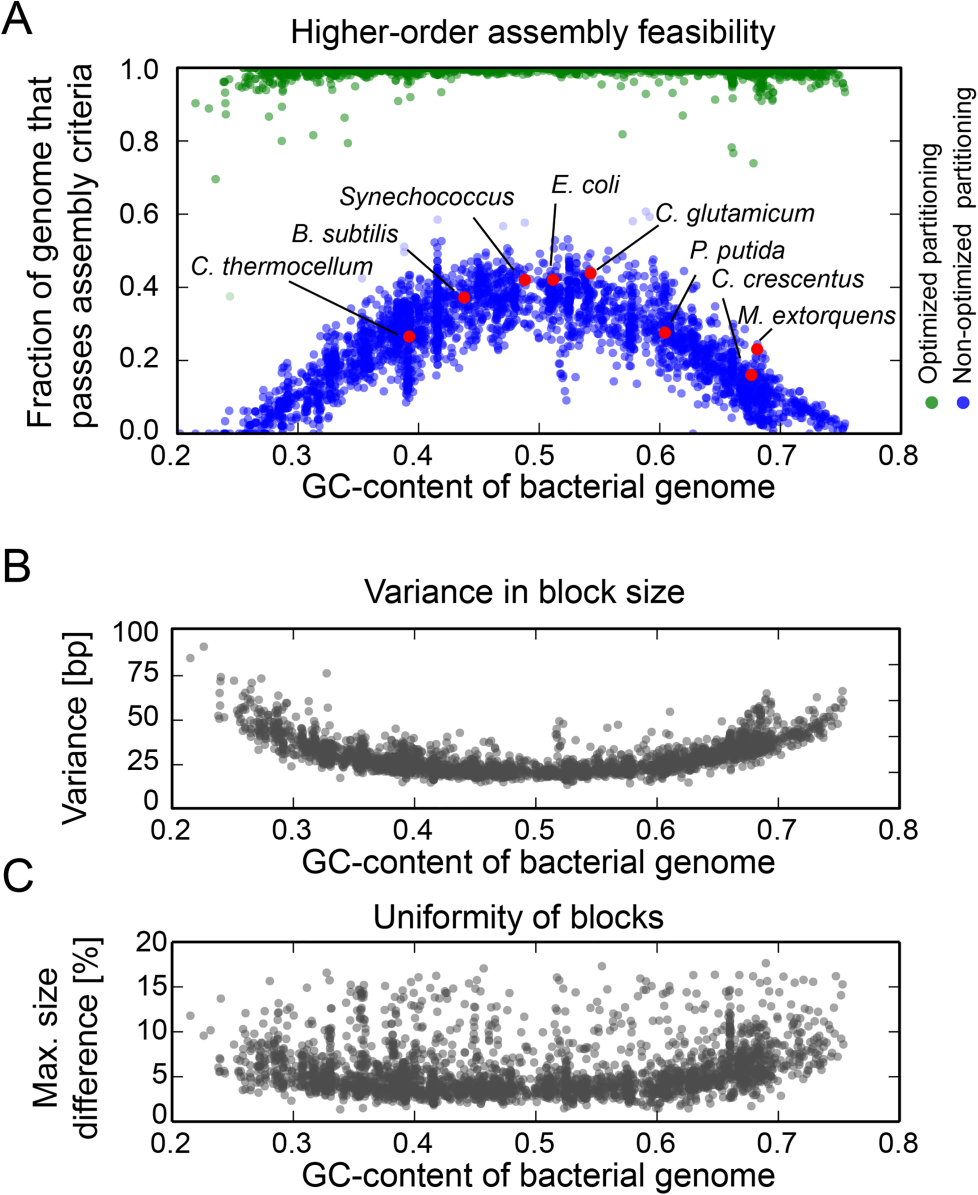
Results from the bioinformatics assembly feasibility analysis across 4967 sequenced bacterial genomes and plasmid sequence deposited at NCBI GenBank database. (A) The fraction of each genome sequence that can be assembled in a risk-free manner into 20 kb genome segments is shown for a non-optimized, equidistant partitioning design (blue) and after computational optimization (green). Commonly used synthetic biology model-organisms are highlighted (red). Subtle repositioning of the overlapping regions between assembly units is sufficient to remove assembly constraints. The introduced size variance (B) and relative size uniformity (C) upon optimization of the 80 bp long overlaps at the block level are plotted as a function of the GC-content. Detailed assembly feasibility statistics are listed in S1 File.

Out of the 11.18 million subblocks generated, 7.8% showed assembly constraints within their sequence overlaps including 1.9% that showed direct and indirect repeats (>8 bp) and 5.9% that contained non-orthogonal overlap sequences, indicating that assembly constraints are prevalent across bacterial genomes (Fig 3). As a general trend, the predicted assembly success rate correlates with the GC content (Fig 3A). However, even for the *E*. *coli* BL21, with a well-balanced GC content of 51.1%, only 37.9% (88/219 20 kb segments) passed our assembly criteria (Fig 3A and Table 2). For the 59.3% of microbial genomes with a GC-content below forty or higher than sixty percent, we observed an even more dramatic picture with less than 17.8% of all sequences amenable to homology based DNA assembly using non-optimized partitioning designs. Among these genomes are several prokaryotic organisms of particular interest for synthetic biology and industrial biotechnology applications that possess difficult-to-assembly genomes (Table 2). The secondary metabolite producing *Streptomyces coelicolor* with a GC-content of 72.5% showed an extremely low predicted DNA assembly success rate of only 6.1% due to a high abundance of non-specific sequence overlaps. Similarly, the cell-cycle model organism *Caulobacter crescentus* [21] showed an assembly success rate of 16.0% (33/206 20kb segments) while for the methanol degrading organism *Methylobacterium extorquens* AM1 only 8.2% (258/281 20 kb segments) can be assembled in a risk-free manner.

**Table 2:** Occurrence of DNA assembly constraints across selected bacterial genomes.

Organism (Genome size)	GC [%]	DNA assembly rate^a^ [%]	Number of assembly constraints		Optimized partitioning, assembly rate^d^ [%]	Block size variance^e^ [bp] upon optimization
			hairpins, repeats^b^	nonspecific overlaps^c^		
*Bacillus subtilis* (4.2 Mb)	44.3	37.2	83	140	100	22.4
*Cornybacterium glutamicum* (4.0 Mb)	54.2	43.8	67	984	98.8	21.1
*Caulobacter crescentus* (4.0 Mb)	67.8	16.0	145	304	100	28.5
*Escherichia coli* (4.6 Mb)	51.1	37.9	110	109	100	20.9
*Pseudomonas putida* (6.9 Mb)	62.0	26.6	207	334	99.4	23.6
*Streptomyces coelicolor* (8.7 Mb)	72.5	6.1	443	908	98.2	39.3
*Synechococcus elongatus* (2.7 Mb)	55.8	22.48	56	123	100	22.5
*Methylobacterium extorquens* (5.5 Mb)	69.1	8.2	238	529	98.9	37.6

^*a*^ Percentage of genome sequences amenable to a four-tier hierarchical assembly into 20 kb segments using equidistant partitioning without optimization of sequence overlaps.

^*b*^ Sum of direct and indirect repeat sequences larger than 8 bp detected within terminal homology regions of equidistant partitioning design.

^*c*^ Total number of non-unique sequence stretches present within multiple terminal homology regions.

^*d*^ Percentage of genome sequences amenable to a four-tier hierarchical assembly into 20 kb segments upon optimization of the partitioning design using the Genome Partitioner algorithm.

^*e*^ Variance of block size upon optimized partitioning in base pair.

### Level of refactoring required to yield a partitioning-optimized genome design

To assess the level of optimization required to streamline bacterial genome sequences for terminal homology based DNA assembly, we partitioned all bacterial GenBank files (downloaded from NCBI) with the Genome Partitioner algorithm. We used standard DNA assembly parameters (see Materials and Methods) and enabled the Genome Partitioner’s built-in algorithm to search for optimal terminal homology regions. We found that after partitioning, 99.4% of all genome sequences passed assembly criteria. The Genome Partitioner algorithm achieves, removal of hairpin, direct repeats and non-specific sequences within overlaps with 99.5%, 98.9% and 94.8% efficiency respectively (S1 File). Uniform size and number of partitioning units is desired to standardize and automate large-scale DNA assemblies. Overall, we observed that for the 80bp and 35bp long overlaps at the block and subblock level, a mean displacement of only 22.6 bp (± 6.3 bp) and 7.6 bp (± 1.9bp) was sufficient to optimize terminal homology regions (Fig 3B and 3C and S3 Fig). Thus, partition-optimized DNA designs require only minor shift within terminal homology regions of assembly units. We concluded that the Genome Partitioner algorithm generates optimized DNA partitioning designs with uniform length, which facilitates standardization and automation of the assembly process

### Assembly of a 20 kb genome segment

**Fig 4:**
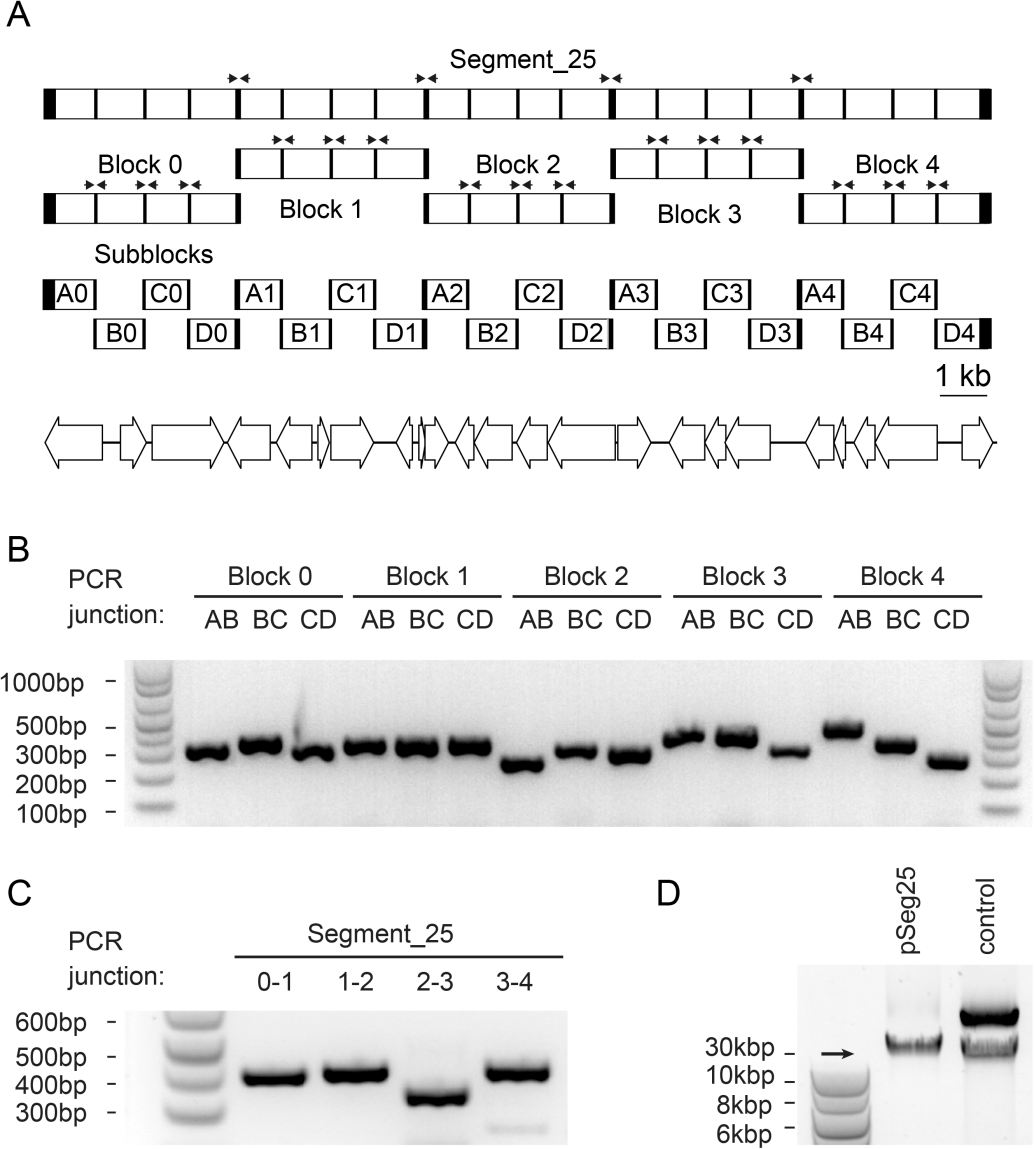
Assembly of a 20 kb long segment 25 from 1 kb subblocks generated by the Genome Partitioner algorithm. (A) Pairs of PCR primers (black arrows above segment and block emblems) generated by the Genome Partitioner algorithm are used to verify assembly junctions. (B) Assembly junctions at the block level are verified by PCR using a set of PCR primer pairs generated by the Genome Partitioner algorithm. (C) Yeast-assembly yields a 20 kb segment from five 4 kb blocks. The assembled segment is verified by PCR amplifying each junction between the different assembly blocks. (D) The correct size of the maintenance plasmid for segment 25 (30 kb) is compared to a supercoiled plasmid control (30 kb) using agarose gel electrophoresis.

To test the assembling efficiency of the DNA units generated by the Genome Partitioner algorithm, we assembled a 20 kb chromosome segment derived from a 774 kb synthetic genome design, we recently compiled from the complete set of essential and fitness genes from *Caulobacter crescentus* identified by high density transposon mutagenesis (TnSeq) [22]. Using the Genome Partitioner web-tool, we partitioned segment 25 from the genome design into five 4 kb DNA blocks that were further subdivided into twenty 1 kb DNA subblocks (Fig 4A). Subblocks generated in a FASTA output file were ordered from a commercial supplier of *de novo* DNA synthesis (Gen9, Inc. Cambridge, MA, USA) and provided as sequence confirmed, plasmid cloned constructs.

Isothermal *in vitro* assembly reactions were used to yield 4 kb DNA blocks from 1 kb subblocks. We started hierarchical assembly by PCR amplification of subblocks from the corresponding maintenance vectors (pG9m-2). Next, the PCR amplified DNA was digested with BbsI type IIS restriction enzyme. Five digestion reactions, each containing a pool of all four corresponding subblocks for a given block were performed for release of subblocks adapter sequences. Using isothermal assembly reactions, the BbsI-digested subblocks pools were ligated into the corresponding blocks and integrated into the maintenance vector pXMCS-2 (Supporting Information and Methods). Subsequently, the resulting plasmids containing the ligated DNA blocks were electroporated into *E*. *coli* DH5α. We verified block assemblies by colony PCR using the Genome Partitioner's automatically designed primer sets that specifically amplify subblock junctions (Table 3). All tested clones (10/10) showed the desired subblock-junction amplicons (Fig 4B) indicating the robustness and reliability of the assembly design generated by the Genome Partitioner algorithm.

**Table 3:** Block assembly success rate from 1 kb subblocks.

Isothermal DNA assembly of 4 kb blocks		Colony number^a^	Assembly success rate^b^
block [0]	subblocks [0–3]	244	2/2
block [1]	subblocks [4–7]	7	2/2
block [2]	subblocks [8–11]	155	2/2
block [3]	subblocks [12–15]	156	2/2
block [4]	subblocks [16–19]	63	2/2
**Yeast assembly of 20 kb segment**			
Segment_25	Blocks [0–4]	230	5/6

^*a*^ Number of transformants recovered

^*b*^ The number of colonies harboring the correct assembly junctions is shown together with the total number of colonies assayed.

Recombineering in yeast was used for the assembly of 4 kb blocks into the 20 kb segment. We purified the plasmids containing the set of DNA blocks needed to assemble the segment and released blocks from the pXMCS-2 backbone by BspQI restriction digestion. Purified digestion mixtures of blocks were used to assemble the segment from the individual blocks into the yeast vector pMR10Y. The correct assembly at the segment level was verified by colony PCRs amplifying each block junction using the Genome Partitioner's automatically designed primers. The PCR verified, yeast-assembled chromosome segment was isolated and electroporated into *E*. *coli* DH5α. The size of the super-coiled plasmid was compared to a previously constructed reference plasmid (pMR10Y::seg_8) [13]. In addition, all block junctions were sequence verified by standard Sanger sequencing (Microsynth AG, Switzerland). In sum, the assembly of the 20 kb test segment indicates that the Genome Partitioner provides a streamlined partitioning strategy to transform genome-scale DNA designs into fabricable synthetic DNA.

## Conclusions

Synthetic Biology as a design and engineering discipline of biologically based parts, devices and systems has the potential to deliver important new applications [23–25], that will improve existing industrial processes [26–30] and advance the design of genetic devices that provide novel treatment strategies for future gene- and cell-based therapies [31,32]. The ability to rapidly program biological functions through streamlined DNA fabrication methods is one of the key technological drivers of innovation in synthetic biology [33]. With recent advances in low-cost DNA manufacturing, what limits the progress of synthetic biology is no longer the scale of gene synthesis but difficulties to efficiently build longer DNA designs that encompass dozens to hundreds of genes for genome-scale biological programs [34,35].

Currently, the majority of available software tools streamline FASTA sequences of individual genes with most of them focusing on optimization yields for recombinant protein expression [36,37], rather than DNA synthesis optimization per se. Here, we have applied the concept of retrosynthesis, a technique for planning and optimizing chemical synthesis routes of complex organic molecules on the partitioning of genome-scale DNA constructs. In so doing, the Genome Partitioner algorithm conceptually decomposes genome-scale DNA designs into simpler building units for the synthesis of which standardized DNA ligation and amplification techniques are known. Thereby, one gradually obtains shorter DNA building blocks, which are ultimately in the size range accessible by low-cost *de novo* DNA synthesis. This process leads to a tree-like design allowing standardized bottom-up genome assembly of genome-scale DNA designs that begins with a panel of several hundreds, sequence-verified 1 kb DNA subblocks.

Finally, the Genome Partition algorithm is not intended to assist in the biological design process of DNA parts. However, the standardized GenBank file format used by the Genome Partitioner is compatible with synthetic biology software tools that do support such biological design [38]. It is important to note that standardization of adaptor sequences is key for streamlined part assembly. As a consequence, internal type IIs restriction sites within the DNA design have to be removed upon synonymous recoding for which several DNA design software tools can be used [13,14,39–41]. Given the minimal level of recoding introduced upon sequence streamlining, it is likely that encoded genetic features and programs retain their functionality. In the rare cases where recoding of endogenous type IIS restriction sites interferes with biological functionality, native sequences can be reintroduced after completion of the genome assembly process by genome editing methods such as CRISPR/CAS.

In summary, the Genome Partitioner web tool presented here enables researchers to build a retrosynthetic roadmap for efficient manufacturing of genome-scale DNA designs. Without the need for specialized computing skills, biologists can submit their DNA designs to a fully automated pipeline that generates and computes the optimal retrosynthetic route for genome-scale *de novo* DNA synthesis and assembly.

### Web tool availability

The Genome Partitioner web tool is available free-of-charge for non-commercial (e.g. academic, nonprofit, or government) use under an ETH Zürich end-user license agreement (EULA). The algorithm is freely accessible for research and non-commercial uses through the web tool. We fully commit to share the source code of the Genome Partitioner under ETH end user license agreement. We also assure long-term utility and open-access on a server cluster for which maintenance and stability of the hosting is guaranteed by ETH Zürich. The Genome Partitioner web tool can be accessed through the public Genome Partitioner website hosted at https://christenlab.ethz.ch/GenomePartitioner. We have deposited a copy of the Genome Partitioner web tool at the ETH Data Archive. The relevant web tools are available within ETH can be found under the following DOI(s), URL(s): DOI: 10.5905/ethz-1007-90, http://doi.org/10.5905/ethz-1007-90. For further information, please refer to the public Genome Partitioner website.

## Supporting information

S1 Fig Overview of the adapter sequence design. (A) Each adaptor sequence provides homology regions (in blue) for recombination-based insertion of assembly units into the corresponding maintenance vector. A restriction endonuclease recognition site (in green) permits release of the synthetic DNA for subsequent higher order assembly. (B) Map of the prefix (5') and suffix (3') adapter sequences. At each assembly tier, the algorithm generates nested adaptor sequences consisting of homology regions for seamless recombination-based *in vitro* and *in vivo* assembly and cloning of adjacent units.
(TIF)

S2 Fig Overview of the web tool partitioning output page. (A) The left panel displays the circular graphic map of the optimized, four-tier hierarchical partitioning design generated by the algorithm. As GenBank test file for partitioning, we have used a sequence-optimized design encompassing the 93.5 kb phototrophic plasmid pSynRL0149 from the *Roseobacter litoralis*. The scheme depicts assembly segments (light and dark pink), followed by blocks (light and dark blue) and subblocks (light and dark green). Segment boundaries adhere to boundaries of biological parts (fourth outermost circle, light and dark grey). The annotated protein coding sequences on forward and reverse strands are shown on the two inner most tacks of the graphic output file. (B) The data output files to be downloaded by the user include an annotated output GenBank file, FASTA files listing the assembly units for each assembly level, log and partitioning parameter files and optional primer lists to validate assembly at each assembly level. (C) A statistical output summarizes the partitioning results obtained for segment, block and subblock levels and provides an overview on statistics of the partitioning design generated.
(TIF)

S3 Fig Effect of partitioning optimization on the uniformity of subblocks. (A) The partitioning analysis was performed across all sequenced bacterial genomes available from NCBI database. The introduced size variance upon optimization of the 35bp long overlaps at the subblock level is plotted as a function of the GC-content of the genome sequence (grey). (B) The relative size difference between the largest subblock and the mean subblock size is plotted for all analyzed genomes as a function of the GC-content of the genome sequence.
(TIF)

S1 File Supplementary data is provided as an Excel file. Worksheet A lists the parameters used by the Genome Partitioner to assess the *de novo* assembly feasibility of all sequenced bacterial genomes. Worksheet B lists the partitioning and assembly analysis using an equidistant partitioning mode without optimization of sequence overlaps between adjacent assembly parts. Worksheet C lists the results obtained from the partitioning and assembly analysis using an optimized partitioning mode with optimization of sequence overlaps between adjacent assembly parts by the Genome Partitioner algorithm.
(XLSX)

S1 Table List of primers used in this study. (DOCX)

S2 Table List of bacterial strains used in this study. (DOCX)
