# Supplementary figures and images for: Genome Partitioner: A web tool for multi-level partitioning of large-scale DNA constructs for synthetic biology applications
Source: PLoS One. 2017 May 22;12(5):e0177234. doi: 10.1371/journal.pone.0177234 (PMC5439662; doi:10.1371/journal.pone.0177234)

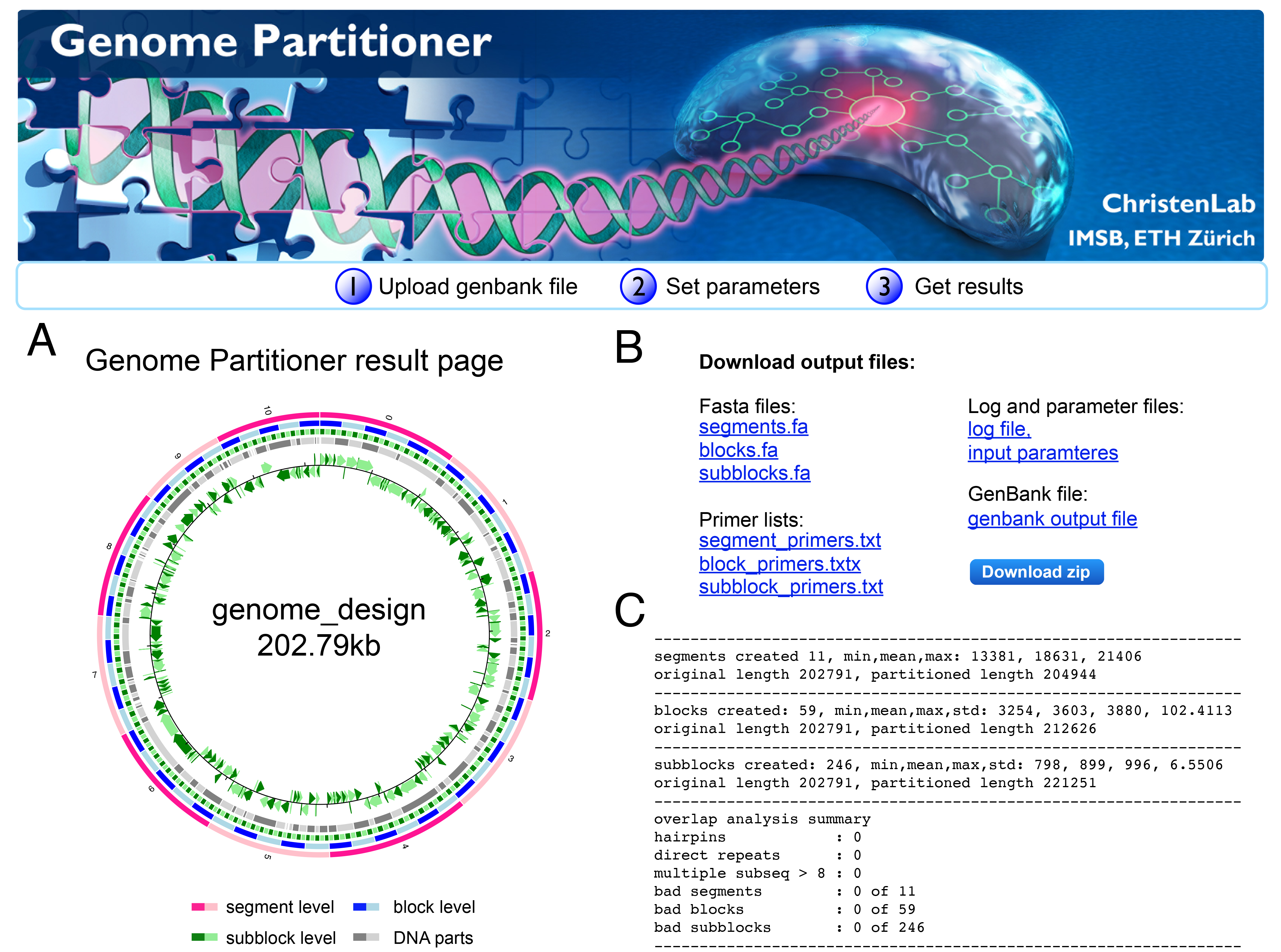

Supplement: S1 Fig — (A) Each adaptor sequence provides homology regions (in blue) for recombination-based insertion of assembly units into the corresponding maintenance vector. A restriction endonuclease recognition site (in green) permits release of the synthetic DNA for subsequent higher order assembly. (B) Map of the prefix (5') and suffix (3') adapter sequences. At each assembly tier, the algorithm generates nested adaptor sequences consisting of homology regions for seamless recombination-based in vitro and in vivo assembly and cloning of adjacent units. (TIF) [file pone.0177234.s001.tif]

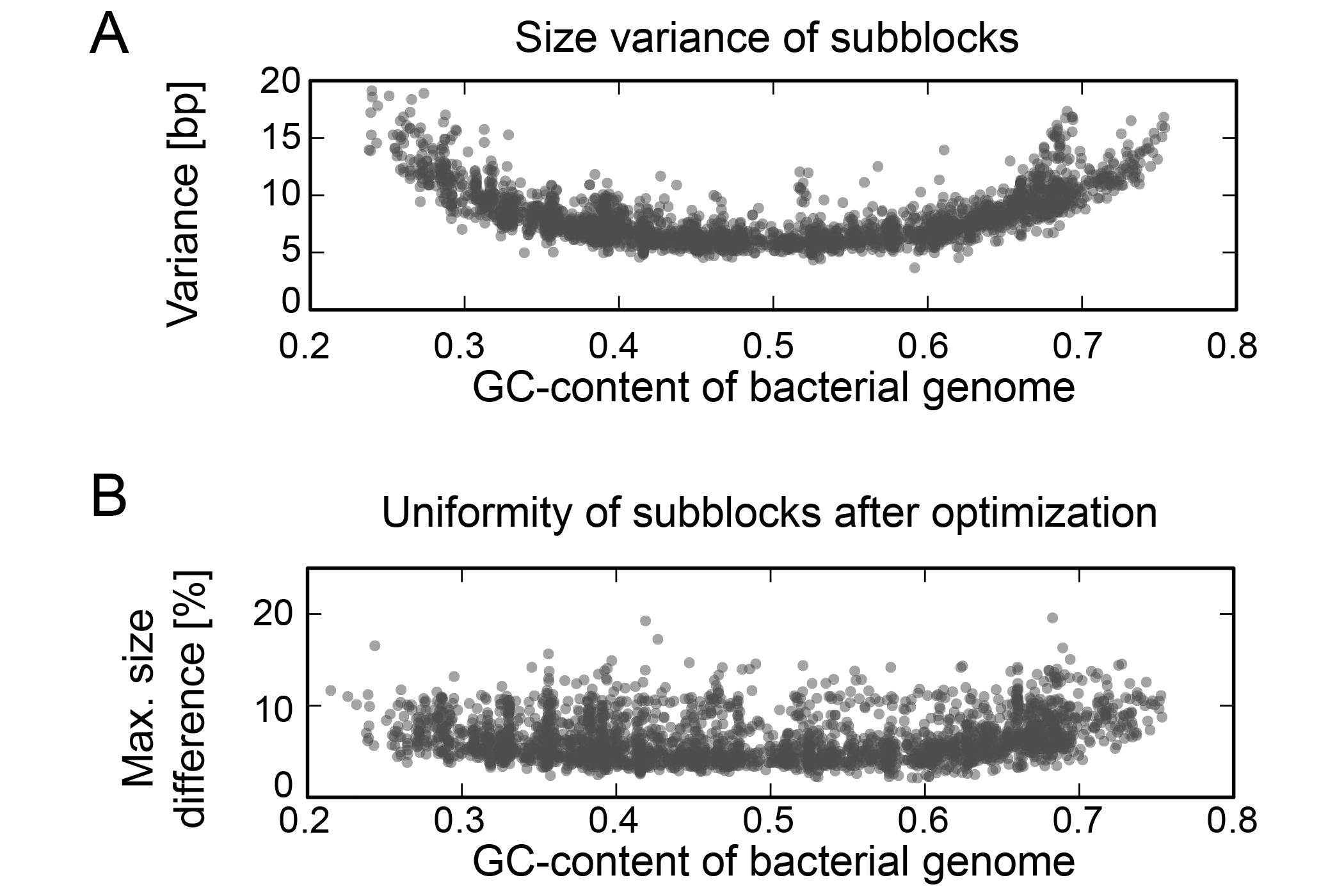

Supplement: S2 Fig — (A) The left panel displays the circular graphic map of the optimized, four-tier hierarchical partitioning design generated by the algorithm. As GenBank test file for partitioning, we have used a sequence-optimized design encompassing the 93.5 kb phototrophic plasmid pSynRL0149 from the Roseobacter litoralis. The scheme depicts assembly segments (light and dark pink), followed by blocks (light and dark blue) and subblocks (light and dark green). Segment boundaries adhere to boundaries of biological parts (fourth outermost circle, light and dark grey). The annotated protein coding sequences on forward and reverse strands are shown on the two inner most tacks of the graphic output file. (B) The data output files to be downloaded by the user include an annotated output GenBank file, FASTA files listing the assembly units for each assembly level, log and partitioning parameter files and optional primer lists to validate assembly at each assembly level. (C) A statistical output summarizes the partitioning results obtained for segment, block and subblock levels and provides an overview on statistics of the partitioning design generated. (TIF) [file pone.0177234.s002.tif]

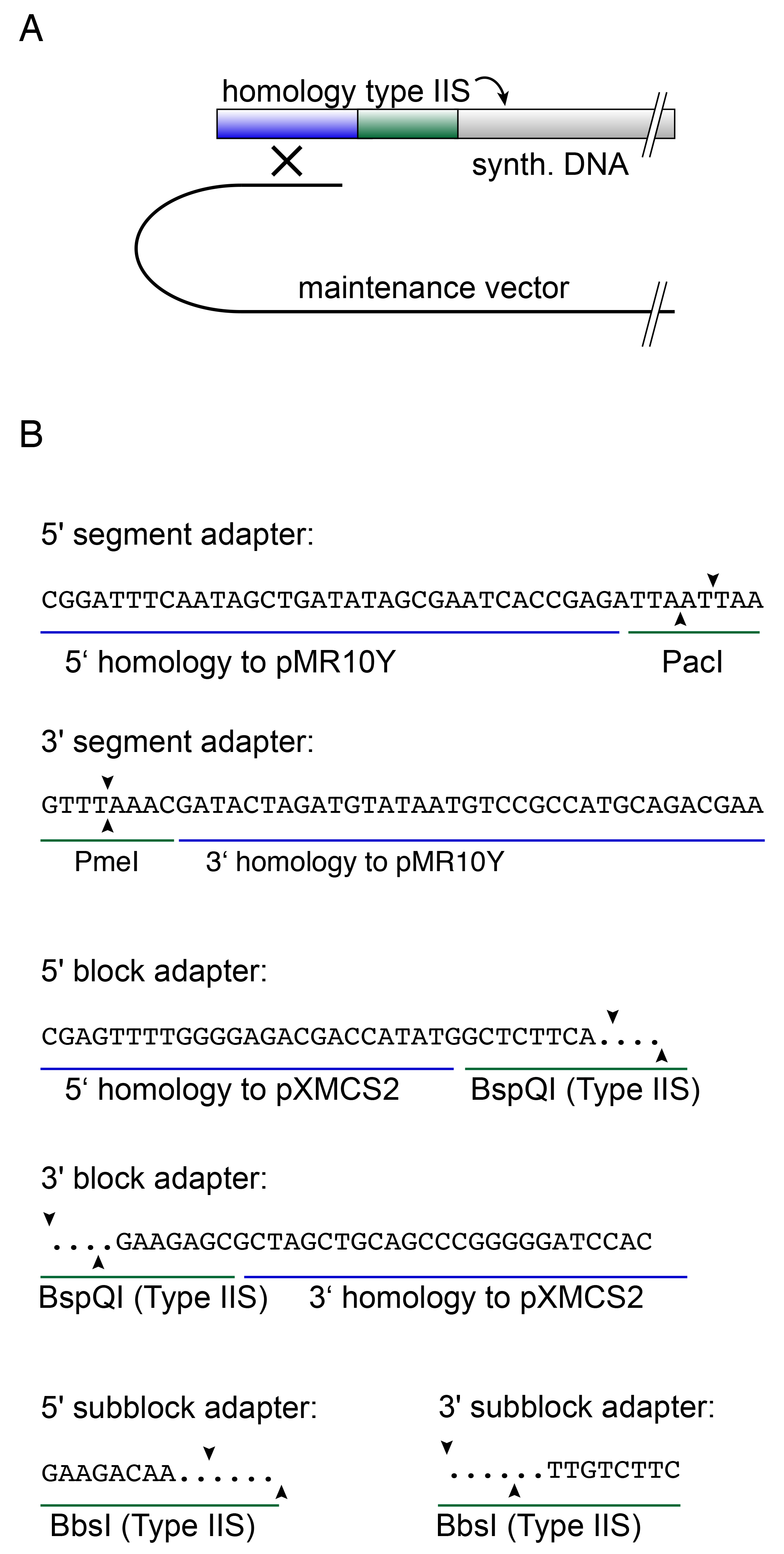

Supplement: S3 Fig — (A) The partitioning analysis was performed across all sequenced bacterial genomes available from NCBI database. The introduced size variance upon optimization of the 35bp long overlaps at the subblock level is plotted as a function of the GC-content of the genome sequence (grey). (B) The relative size difference between the largest subblock and the mean subblock size is plotted for all analyzed genomes as a function of the GC-content of the genome sequence. (TIF) [file pone.0177234.s003.tif]
