# Supplementary material for: Genome Partitioner: A web tool for multi-level partitioning of large-scale DNA constructs for synthetic biology applications
Source: PLoS One. 2017 May 22;12(5):e0177234. doi: 10.1371/journal.pone.0177234 (PMC5439662; doi:10.1371/journal.pone.0177234)
Supplement: S1 Table — (DOCX) [file pone.0177234.s005.docx]

**Table S1:** Primer List

| Primer ID | Name | Sequence |
| --- | --- | --- |
| 1 | >pG9m2_MCS_fw | GTGAAGGTGAGCCAGTGA |
| 2 | >pG9m2_MCS_rv | GAAAGTCAAAAGCCTCCG |
| 3 | >subbl_ov_0_1_fw | CCTGCACAGGCTCGACGATG |
| 4 | >subbl_ov_0_1_rv | CGTTCGCCGACGTGGTGTTC |
| 5 | >subbl_ov_1_2_fw | GCCAAGCAACTAGGCGGCGT |
| 6 | >subbl_ov_1_2_rv | GCGACGACCGCAGAAGGTGA |
| 7 | >subbl_ov_2_3_fw | CCTGTCAGGTGCTGGTCTGG |
| 8 | >subbl_ov_2_3_rv | GGCGATCCGAGACGAAGTCG |
| 9 | >subbl_ov_4_5_fw | CCACACCCATCATGCGCACG |
| 10 | >subbl_ov_4_5_rv | TCCGCTGGTGATCGACCTGG |
| 11 | >subbl_ov_5_6_fw | CGCGTGCTATAGGCGAGCCA |
| 12 | >subbl_ov_5_6_rv | GCGCATCGGCTTCTACAGCG |
| 13 | >subbl_ov_6_7_fw | ACGCACGCTCCCCTGACCAT |
| 14 | >subbl_ov_6_7_rv | GGCTCTGCGCTGTTGAGGTC |
| 15 | >subbl_ov_8_9_fw | GCCATAGCTGCCCCAAGAGC |
| 16 | >subbl_ov_8_9_rv | GTCGTGCTTTGGGGCGTACG |
| 17 | >subbl_ov_9_10_fw | CTCCGGAACGGTCGCTTGGA |
| 18 | >subbl_ov_9_10_rv | TGGTTGTCACCGACGGCGGT |
| 19 | >subbl_ov_10_11_fw | CGGCGCCGATATTGGCCTTC |
| 20 | >subbl_ov_10_11_rv | CGGCGCGGTTGTCGAACAGT |
| 21 | >subbl_ov_12_13_fw | CTCTCGCGGATCGGTCCCTT |
| 22 | >subbl_ov_12_13_rv | TCGACTCCGGGGCGTTTTCC |
| 23 | >subbl_ov_13_14_fw | ACCCTTCTTGCGACGTGGGC |
| 24 | >subbl_ov_13_14_rv | TCGAAGTGAACCTGCCGCCG |
| 25 | >subbl_ov_14_15_fw | GCTTGTTGAGCGCGGCGAAC |
| 26 | >subbl_ov_14_15_rv | TTTTGCCCAGGACGCCGCAG |
| 27 | >subbl_ov_16_17_fw | CAGATAGCCGCGAGCGTACG |
| 28 | >subbl_ov_16_17_rv | GCGATGTGACCAGCGTCCAG |
| 29 | >subbl_ov_17_18_fw | TCGATGTCGACGGCGGTCAG |
| 30 | >subbl_ov_17_18_rv | ATCCACAACGCCGCCTGCGA |
| 31 | >subbl_ov_18_19_fw | TCAGCATGATCCGGGCGTGC |
| 32 | >subbl_ov_18_19_rv | GTCGGTCGCAGGATGACGCT |
| 33 | >block_ov_0_1_fw | GACGCGGTTATCGATGGCGA |
| 34 | >blockl_ov_0_1_rv | GGTTTCGGGCGGTTGTCCAT |
| 35 | >block_ov_1_2_fw | AGCAGCATGGCGGGGAAGTT |
| 36 | >blockl_ov_1_2_rv | CCACCTACAGCTGCTTGCCA |
| 37 | >block_ov_2_3_fw | CCCACCACGACAATGATGCG |
| 38 | >blockl_ov_2_3_rv | CCACAAGATCTGGCGCGGTA |
| 39 | >block_ov_3_4_fw | ACTGAGCTACCCAGGCATCC |
| 40 | >blockl_ov_3_4_rv | TCGAGACGAAGGTCGGCTTC |
