# Supplementary material for: Genome Partitioner: A web tool for multi-level partitioning of large-scale DNA constructs for synthetic biology applications
Source: PLoS One. 2017 May 22;12(5):e0177234. doi: 10.1371/journal.pone.0177234 (PMC5439662; doi:10.1371/journal.pone.0177234)
Supplement: S2 Table — (DOCX) [file pone.0177234.s006.docx]

**Table S2**: Bacterial Strains and Plasmids

| Strain, plasmids | Description | Reference or source |
| --- | --- | --- |
| BC3744 | *E. coli* (DH5α), pXMCS-2::block0 | this work |
| BC3745 | *E. coli* (DH5α), pXMCS-2::block1 | this work |
| BC3746 | *E. coli* (DH5α), pXMCS-2::block2 | this work |
| BC3747 | *E. coli* (DH5α), pXMCS-2::block3 | this work |
| BC3748 | *E. coli* (DH5α), pXMCS-2::block4 | this work |
| BC3347 | *S. cerevisiae* (VL6-48N), MAT α, *his3-D200*, *trp1-Δ1*, *ura3-Δ1*, *lys2*, *ade2- 101*, *met14*, psi+cir° | [1] |
| pMR10Y | pMR10 plasmid with ARS/CEN, URA3 | [2] |
| pMR10Y::seg_8 | pMR10Y with 19 kb chromosome segment_8 | [2] |
| pXMCS2 | narrow host range plasmid with pMB1 origin | [3] |

References

1. Larionov V, Kouprina N, Solomon G, Barrett JC, Resnick MA. Direct isolation of human BRCA2 gene by transformation-associated recombination in yeast. Proc Natl Acad Sci USA. National Academy of Sciences; 1997;94: 7384–7387.

2. Christen M, Deutsch S, Christen B. Genome Calligrapher: A Web Tool for Refactoring Bacterial Genome Sequences for de Novo DNA Synthesis. ACS Synth Biol. American Chemical Society; 2015;4: 927–934. doi:10.1021/acssynbio.5b00087

3. Thanbichler M, Iniesta AA, Shapiro L. A comprehensive set of plasmids for vanillate- and xylose-inducible gene expression in Caulobacter crescentus. Nucleic Acids Res. 2007;35: e137–e137. doi:10.1093/nar/gkm818
